# Infection with *Babesia bovis* alters metabolic rates of *Rhipicephalus microplus* ticks across life stages

**DOI:** 10.1186/s13071-024-06645-3

**Published:** 2025-03-01

**Authors:** Kayla N. Earls, Karen Poh, Massaro Ueti, Kennan Oyen

**Affiliations:** 1 Department of Veterinary Microbiology and Pathology, Washington State University, Pullman, WA 99164-7040 USA; 2 Animal Diseases Research Unit, United States Department of Agriculture, Agricultural Research Service, 3003 ADBF, Pullman, WA 99164-6630 USA

**Keywords:** Flow-through respirometry, Transovarial transmission, Protozoan pathogen, Cattle fever tick, Arthropod vector, Metabolic rate

## Abstract

**Background:**

Metabolic responses to infection differ based on arthropod and pathogen. Increased metabolic rates can result in faster depletion of energetic resources, and decreases may allow for energy conservation. *Babesia bovis* is a protozoan pathogen transmitted by the cattle fever tick, *Rhipicephalus microplus*. Adult female ticks acquire *B. bovis* by feeding on an infected animal. *Babesia bovis* undergoes development and invades the ovaries where it is transmitted transovarially to tick offspring. The effects of infection on *R. microplus* metabolic rate are not well studied.

**Methods:**

We tested the hypothesis that *R. microplus* infected with *B. bovis* would have altered metabolic rates (volume of carbon dioxide [VCO_2_]) across life stages using flow-through respirometry. Replete females from either an infected or naïve calf were measured across 3 days to determine differences in VCO_2_. Hemolymph smears were used to categorize the number of *B. bovis* kinetes present in the hemolymph of replete females during egg oviposition. The VCO_2_ for groups of their offspring were measured twice as eggs and once as larvae. The number of individuals and successfully hatched larvae in each group were enumerated at the end of the experiment to determine the average VCO_2_ per individual.

**Results:**

Infected replete females have decreased VCO_2_ while their offspring have increased VCO_2_ at the egg and larval stages. Interestingly, replete females had a 25% reduction in body mass compared to uninfected female tick controls. Uninfected larvae were twice as likely to hatch than larvae from infected replete female ticks.

**Conclusions:**

VCO_2_ varied between control and infected ticks depending on life stage. Infected replete females had lower VCO_2_ and body mass while their offspring had higher VCO_2_ than their control counterparts. Higher larval VCO_2_ may promote earlier questing and a shorter lifespan. Changes in metabolic and hatch rates have implications that may promote disease spread.

**Graphical Abstract:**

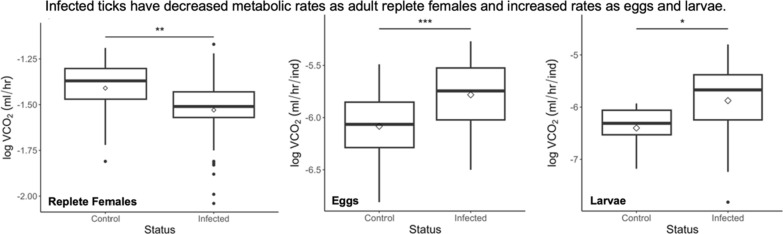

**Supplementary Information:**

The online version contains supplementary material available at 10.1186/s13071-024-06645-3.

## Background

Respiration can be used as a proxy for energy consumption [3]. A reduction in the metabolic rate allows for long-term energy conservation, while an increase would result in a faster depletion of resources [4]. For long-lived species with low metabolic rates, shifts in metabolic rates influence life history traits, such as longevity and behavior [5–8]. Metabolic changes in vectors that go long periods without feeding, such as ticks, could impact disease spread as individuals need to seek out hosts before energy stores are too depleted [5, 9, 10]. The unique relationship between vector-borne pathogens and their invertebrate intermediate host involves an evolutionary arms race that can affect changes in metabolic resources [11]. Whether infection exerts an energetic cost or benefit to its vector could have major implications for understanding the spread of disease within a population. Suppression of metabolism in the presence of infection stress may allow for energy conservation [4, 12], while an increase may indicate the pathogen sequestering resources and/or the need for energy to fight infection [13, 14].

Measuring whole-organism metabolic rates can show changes in energy consumption in response to immune challenges. General induced immune responses, such as those resulting from implantation and wounding, can increase metabolic rates in a wide range of insect taxa including lepidopterans, coleopterans, orthopterans, and blattodeans [14, 15]. However, shifts in metabolic rate due to specific pathogens and viruses have mixed responses across species. Infection with *Drosophila* C virus (DCV) causes decreased metabolic rates in *Drosophila melanogaster* [16]. Similar responses are seen in mosquitoes (*Aedes aegypti*) infected with poultry malaria (*Plasmodium gallinaceum*) and crustaceans (*Litopenaeus vannamei* and *Callinectes sapidus*) infected with the bacteria *Vibrio campbellii* [17–20]. Contrarily, the hemipteran *Diaphorina citri* had increased metabolic rates when infected with the bacterium *Candidatus* Liberibacter asiaticus [21]. Mosquitoes (*Ae. aegypti*) infected with virulent *Wolbachia pipientis* also had increased metabolic rates in males and females [22]. Differences in metabolic responses demonstrate the need to measure more species and pathogens to understand underlying patterns associated with the energetics of infection.

*Babesia bovis* is a protozoan pathogen that infects bovines and is transmitted by ticks. *Babesia bovis* is transmitted transovarially, allowing the pathogen to persist across generations and life stages [23–25]. Through this process of transmission, *B. bovis* as kinetes invade the ovaries and eggs inside replete adult females [23, 26, 27]. The subsequent generation of offspring acquire *B. bovis* from the infected maternal female tick. The larval stage transmits *B. bovis* after the sporozoites invade the salivary glands and are transferred to an animal during feeding [23, 26, 27]. *Rhipicephalus microplus* is the main vector for *B. bovis*. Eradication and management practices have been utilized for nearly a century to control the spread of *B. bovis* by *R. microplus* in the United States [28–30]. *Babesia bovis* causes anemia, jaundice, increased respiration, reduced fertility, and abortions in pregnant cattle [28]. Control approaches are primarily focused on tick eradication using pesticides; however, the emergence of several pesticide-resistant strains of *R. microplus* has increased the urgency to develop novel methods of control for *B. bovis* and *R. microplus* [31–34]. Clarifying how *B. bovis* influences tick physiology and energetics may shed light on new methods for control, including reducing parasitemia in the tick to non-transmissible levels. The effect of *B. bovis* infection may have long-term effects on tick physiology across life stages driving evolutionary shifts in mutualistic or antagonistic relationships between the vector and pathogen.

Adult ticks, including *R. microplus*, have exceptionally low metabolic rates and utilize discontinuous gas exchange during times of low energy demand [9, 35–37]. Ticks survive long periods without feeding, relying on energy conservation until seeking a host when energy demands shift [9]. Changes in metabolic rates due to infection could influence energetic stores, questing behavior, and pathogen transmission; therefore, it is essential to measure how *R. microplus* respiratory physiology changes across life stages after pathogen infection. We hypothesize that *B. bovis* infection alters metabolic rates across life stages in *R. microplus*. To test this hypothesis, metabolic rates of infected and uninfected ticks were compared across three life stages: adult females, resulting eggs, and hatched larvae. Adult *R. microplus* were allowed to feed on a calf that was either infected with *B. bovis* or naïve. Metabolic rates (volume of carbon dioxide [VCO_2_]) of replete females were measured for 3 days following drop-off. Their offspring were measured in groups at the egg and larval stages.

## Methods

### Cattle and ticks

One splenectomized Holstein steer calf was used for the *B. bovis* acquisition experiment, and one spleen-intact Holstein steer calf was used as the control for infected and uninfected ticks, respectively. A splenectomized calf was used for the acquisition experiment to maximize the opportunity for successful *B. bovis* infection. The calf was splenectomized at least 4 weeks before the start of the experiment. Prior to the start of the experiment, both calves were confirmed healthy, based on a physical exam, complete blood count, and serum chemistry panel, and were negative for *B. bovis* via nested polymerase chain reaction (nPCR) targeting the *BBOV_I002220* gene [38].

Laboratory-colonized *R. microplus* of the La Minita strain were used in the experiment. These originated from an outbreak in Starr County, Texas, in 1996 and have been reared as a colony without the addition of new field-collected ticks [39, 40].

### Acquisition feeding of *R. microplus* ticks

The tick acquisition experiment followed a similar protocol as reported by Poh et al. [41]. Briefly, a splenectomized calf (C1833) was inoculated with a *B. bovis* S74-T3Bo blood stabilate (10^7^ *B. bovis*-infected erythrocytes) that was rapidly thawed, mixed with 5 ml Puck’s saline-G and 10% newborn bovine serum (NBS), and then administered to the calf intravenously through the jugular vein [42–45].

Ten days prior to inoculation, approximately 20,000 hatched larvae were added to stockinette patches on C1833 and allowed to feed through repletion as adults during peak parasitemia. By synchronizing peak parasitemia and tick feeding repletion, this increased the probability of successful acquisition of the parasite in the ticks. At 11 days post-inoculation, the animal reached peak parasitemia, coinciding with the collection of replete females, and the acquisition experiment was terminated. From this acquisition feeding, 32 replete, infected females that were fully engorged at the termination of the experiment were collected for this experiment.

To confirm infection and classify infected ticks, kinetes were stained and enumerated in the hemolymph using light microscopy [46, 47]. Replete females were individually stored in 24-well plates and incubated at 26 °C and 93% relative humidity with a 12 h:12 h light/dark (L:D) cycle, except for when they were inside the respirometer. *Babesia* *bovis* kinetes differentiate in the tick after approximately 8–9 days. Following differentiation, hemolymph was collected by snipping the distal leg segment onto a glass slide, Giemsa-stained, and examined using a light microscope [46, 47]. Ticks that fed on an infected calf were classified based on Howell et al. [25] with the following categories: undetectable, 1–5 kinetes, 5–10 kinetes, and > 10 kinetes.

### Flow-through respirometry setup

Room air from a ventilation duct was pulled through two columns of drierite and ascarite to scrub out water and CO_2_ using a pump (S-S4 Sub-Sampler, Sable Systems). The flow rate of incurrent air was controlled by the subsampler controlling the amount of air entering a humidity chamber and MAVEn-FT (Sable Systems, Las Vegas, NV, USA). The pump ran at 1500 ml/min at 50% for replete females and 500 ml/min at 30% for eggs and larvae. A humidity chamber was added with 500 ml of distilled water to prevent desiccation.

The flow rate was divided amongst 16 animal chambers and a baseline so that each chamber received a continuous 50 ± 0.1 ml/min of oxygen for replete females and 5.0 ± 0.1 ml/min for eggs and larvae. Each chamber was measured for 30 min with a 10-min baseline between each chamber to stabilize CO_2_ readings. Excurrent CO_2_ (parts per million) was measured using an analyzer (LI-850, LI-COR Biosciences, Lincoln, NE, USA) connected via analog to the MAVEn-FT. The CO_2_ analyzer was zeroed before trials and spanned using 50 ppm CO_2_. The rate of CO_2_ emission (VCO_2_, ml/h) was calculated using the following formula [35]:

$${\dot{\text{V}}\text{CO}}_{2} \left(\frac{ml}{hr}\right)=\left({CO}_{2}\left(ppm\right)\div \text{1,000,000}\times chamber\, flow\, rate \left(\frac{ml}{min}\right)\times 60 mins\right)-baseline\left(\frac{ml}{hr}\right).$$

VCO_2_ for eggs and larvae were calculated by dividing absolute VCO_2_ (ml/h) by the number of individuals measured in each chamber.

Temperature (°C), light (lux), humidity (%), and air pressure (kPa) were continuously measured every second during trials. All trials ran at room temperature (21.7 ± 0.12 °C) with constant light (9.94 ± 0.06 lx). The average room humidity was 17.9 ± 1.80% relative humidity, while barometric pressure was 92.9 ± 0.16 kPa.

### Replete females

Female *R. microplus* (*n* = 32) that fed on an infected calf were divided into two groups to be tested in the MAVEn-FT. Female *R. microplus* (*n* = 10) that fed on an uninfected calf were also measured separately. Individual replete females were placed in a 30 ml glass chamber with rubber stoppers to measure CO_2_ output using flow-through respirometry. Each tick was measured once every 24 h for three consecutive days starting on day 1 post-drop-off. Mass (g) was measured to the nearest 0.01 mg using an analytical balance (Sartorius QUINTIX224-1S, Göttingen, Germany) before and after measurements each day.

### Eggs and larvae

**Fig. 1:**
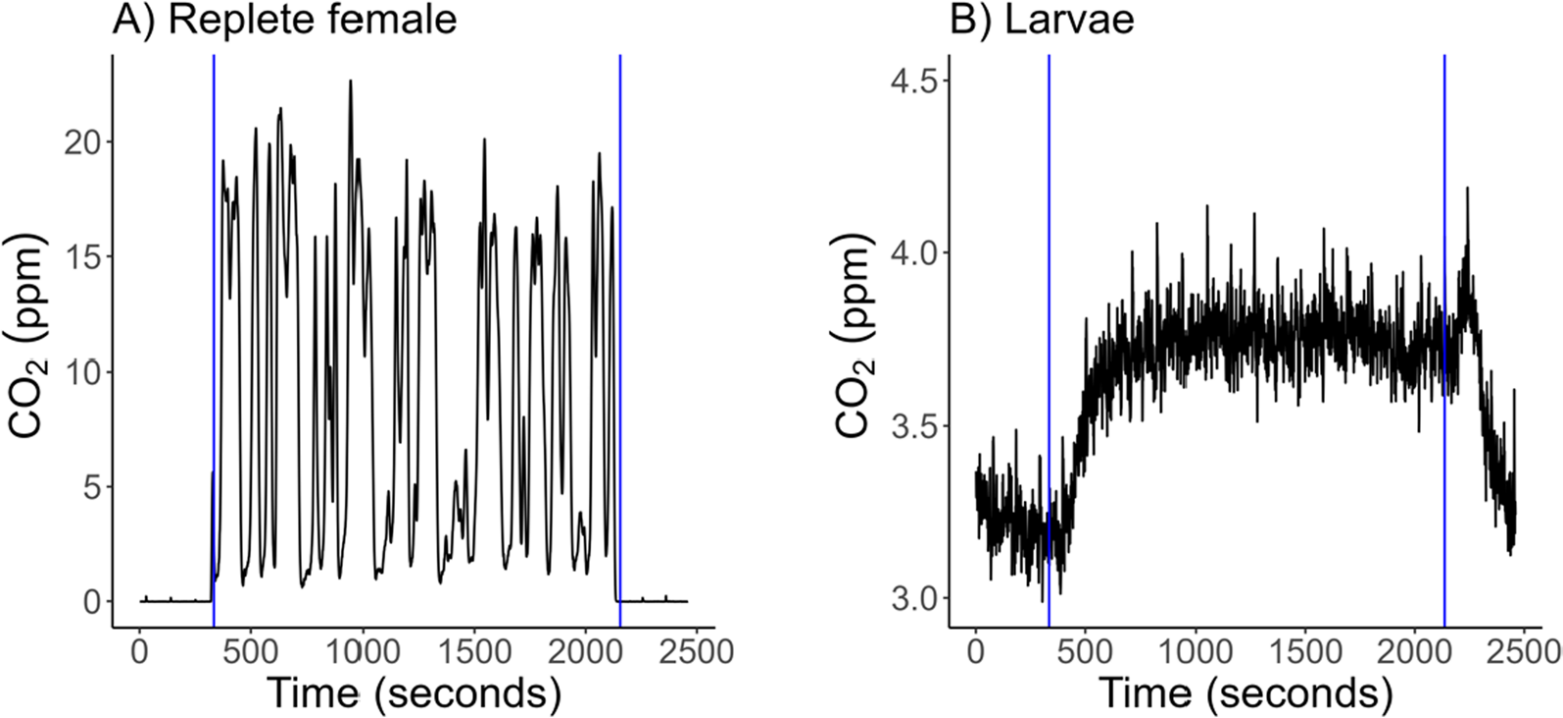
Raw CO_2_ (ppm) output example from replete females (**A**) and larvae (**B**). Blue vertical lines indicate the start and stop of baseline and chamber readings. Replete females actively respired for the entire 30-min chamber readings. Eggs and larvae were measured in groups of individuals because CO_2_ output was not detectable for a single tick

A preliminary experiment was performed to determine the minimum egg mass size required to generate a measurable CO_2_ signal. Egg mass size was determined visually, and the total offspring in the mass were counted after larvae were measured and frozen at −20 °C. While replete females had a cyclic ventilation pattern (Fig. 1A), offspring had a plateau shape (Fig. 1B). The presence of a plateau helped indicate the minimum egg mass size for CO_2_ detection. More than 20 offspring were needed in each group to detect changes in CO_2_.

Females were allowed to lay eggs in a 24-well plate for 3 days before collection of a subsample of eggs. Groups of eggs were placed in a 2 cm coffee straw with fine mesh secured on both ends. Offspring remained in these straws for the entirety of the experiment so they could be measured multiple times as eggs and larvae. Groups of offspring were measured three times for 30 min over a 32-h window. The flow rate of CO_2_-free air into the chambers was 5 ml/min. A baseline measurement was read between each animal chamber containing offspring for 10 min. The first 5 min of the baseline and chamber readings were removed due to a lag effect created by low flow rates. Excurrent CO_2_ was measured by the LI-850 in ppm. Straws containing *R. microplus* were stored in a glass petri dish inside an incubator at 26 °C and 93% relative humidity with 12:12 L:D (same conditions as the replete females) when not in the respirometry system.

Eggs were measured on weeks 1 and 2 post-oviposition. Larvae were allowed to hatch inside the straws and were measured 4 weeks after hatching to allow for exoskeleton hardening. Larvae were visually inspected under a microscope before and after entering the MAVEn animal chambers to document mortality. After larvae were measured, straws were frozen at −20 °C. After at least 24 h, straws were disassembled to count the number of larvae. Offspring were categorized as either successfully hatched larvae or unhatched/dead eggs. The metabolic rate for larvae was divided by the number of offspring that successfully hatched.

### Statistical analysis

All means are presented as standard error (SEM), and significance was determined by *P* < 0.05. All statistical analyses were performed in R (version 4.3.0; R Core Team, 2023) using the following packages: *lme4*, *nlme*, and *emmeans*. Data processing and visualization were performed using *dplyr*, *cowplot*, and *ggplot2*. VCO_2_ and mass were log-transformed for analyses. Overall differences in absolute VCO_2_, mass-specific VCO_2_, and mass were compared using generalized linear mixed-effect models (*nlme* package) for replete females. Only females that laid eggs were included in the analyses. Absolute and mass-specific VCO_2_ of infected and uninfected replete females were compared using linear mixed models with day measured and mass as fixed effects and female ID as a random effect to control for repeated measures. Differences in VCO_2_ on specific days were compared using a *t*-test (e.g., infected vs. uninfected compared only on day 1). Linear regressions were performed for both infected and uninfected ticks to compare metabolic scaling relationships with body size for replete females.

Eggs and larvae were measured three times during the 32 h they were in the MAVEn and were included in linear mixed-effect models as a random effect to account for repeated measures. Hatching success was compared using a binomial generalized linear mixed-effect model (*lme4* package) with tick ID as a random effect. The binomial response variable was either dead or alive per individual tick offspring. All non-significant factors were removed from the final models. Tukey post hoc comparisons (*emmeans* package) were used to detect significant differences in VCO_2_ between infection status across kinete categories. An analysis of covariance (ANCOVA) determined whether the regressions of infected and uninfected ticks were significantly different from each other.

## Results

### Replete females

**Fig. 2:**
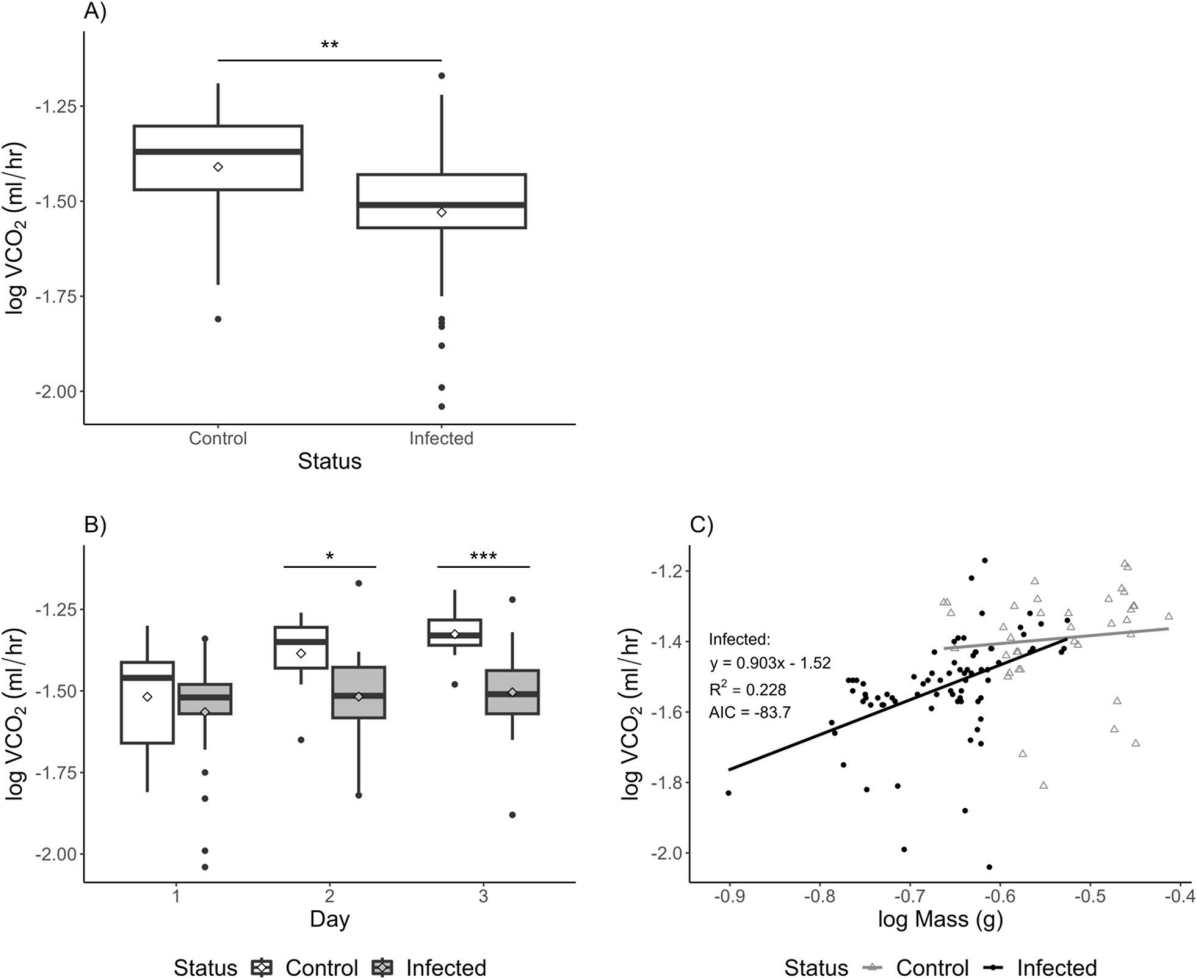
Comparison of absolute VCO_2_ between replete females that fed on either an uninfected calf or a calf infected with *B. bovis*. **A** Control ticks had higher overall VCO_2_ than infected ticks. **B** Replete females were measured across 3 days. Control ticks had higher VCO_2_ on days 2 and 3. Linear mixed-effect model results can be found in Table 2. **C** There were no significant differences between control (gray open triangles) and infected slopes (black closed circles; ANCOVA; *F*_1,111_ = 1.32, *P* = 0.253). However, infected ticks had a significant positive linear relationship with body mass and VCO_2_ (linear regression; *F*_1,71_ = 22.3, *P* < 0.0001). Significant differences are denoted by * < 0.05, ** < 0.01, and *** *P* < 0.001. Extending lines represent the 25th to 75th percentiles of the data. Points beyond are considered outliers

Overall, infected females had lower metabolic rates than control females (Fig. 2A; Tables 1, 2). The day that females were measured was a significant factor in determining metabolic rate (Tables 1, 2; Fig. 2B). On day 1, metabolic rates for control and infected ticks were not different from each other; however, on days 2 and 3, control ticks had higher metabolic rates than infected ticks (Fig. 2B). For control *R. microplus*, day 1 was significantly lower than days 2 and 3 (linear mixed-effect model; *F*_2,18_ = 8.01, *P* = 0.003). The metabolic rate did not change over the 3 days for the infected ticks (linear mixed-effect model; *F*_1,48_ = 2.22, *P* = 0.119).

**Table 1:** VCO_2_ averages with SEM across life stages

Day	Treatment	Average (μl/h) ± SEM
Replete females		
Overall	Control	40.94 ± 2.151
	Infected	31.26 ± 1.164
1	Control	23.42 ± 3.697
	Infected	29.11 ± 1.856
2	Control	42.53 ± 3.139
	Infected	32.01 ± 2.197
3	Control	47.86 ± 2.747
	Infected	32.74 ± 1.998
*Eggs*		
	Control	0.001 ± 0.0001
	Infected	0.002 ± 0.0001
*Larvae*		
	Control	0.0005 ± 0.00009
	Infected	0.003 ± 0.0008

**Table 2:** Metabolic rate results for mixed-effect models comparing control and infected *R. microplus* ticks

	Model term	Estimate	Std error	*t*-value	*P*-value
Replete females					
Absolute VCO_2_	Intercept	−1.52	0.042	−36.5	*P* < 0.0001
	Status (infected)	−0.10	0.030	−3.41	*P* = 0.0011
	Day	0.05	0.013	3.55	*P* = 0.0007
Eggs					
VCO_2_ per individual	Intercept	−6.08	0.059	−103	*P* < 0.0001
	Status (infected)	0.30	0.057	5.27	*P* < 0.0001
Larvae					
VCO_2_ per individual	Intercept	−6.40	0.152	−42.2	*P* < 0.0001
	Status (infected)	0.52	0.200	2.63	*P* = 0.0126

Tick ID was used as a random effect for replete female analysis. Replicate was used as a random effect for egg and larvae analyses

Females were categorized based on hemolymph smears measuring the number of visual kinetes. VCO_2_ of kinete categories was compared across the 3 days (Supplemental Fig. 3; Supplemental Tables 1 and 2). While kinete category and day had a significant effect on VCO_2_ (Supplemental Table 2), differences were primarily driven by the control treatment and > 10 kinete category. Each kinete category was analyzed separately to determine changes over the 3 days. Only the control treatment and > 10 kinetes had significant increases in metabolic rates after day 1 (Supplemental Table 2). Kinete categories were not significantly different from each other when analyzing each day separately (Supplemental Table 2).

**Fig. 3:**
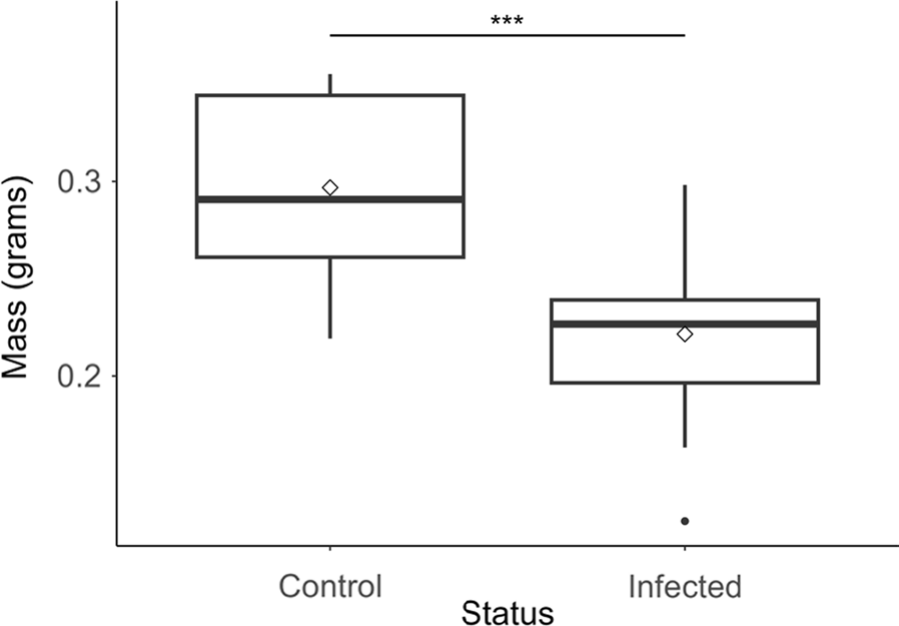
Replete females that fed on an infected calf weighed approximately 0.075 g less than control ticks (linear mixed-effect model; *F*_1,74_ = 168, *P* < 0.0001). Significant differences are denoted by * < 0.05, ** < 0.01, and *** *P* < 0.001. Extending lines represent the 25th to 75th percentiles of the data. Points beyond are considered outliers

Replete females that fed on an infected calf (0.222 g ± 0.004) had approximately 25% lower body masses than control ticks (0.297 g ± 0.008; linear mixed-effect model, *df* = 74, *t* = −13.0, *P* < 0.0001; Fig. 3). Mass did not change across the 3 days females were measured (linear mixed-effect model; *df* = 73, *t* = −1.06, *P* = 0.292). Females digested their blood meal at this time, while they produced eggs which may explain no changes in mass over time. Mass was a significant factor in determining VCO_2_ in females that fed on an infected calf with larger females producing more CO_2_ than smaller individuals (Fig. 2C; linear regression; *F*_1,71_ = 22.3, *P* < 0.0001). Regardless of body mass, control *R. microplus* had similar VCO_2_ (Fig. 2C; linear regression; *F*_1,36_ = 0.435, *P* = 0.514). The linear regression comparing body mass and VCO_2_ of infected ticks did not significantly differ from the linear regression generated by control ticks (ANCOVA; *F*_1,111_ = 1.32, *P* = 0.253).

### Eggs and larvae

Metabolic rates for individual eggs and larvae were undetectable. Therefore, clusters of eggs were measured, and the metabolic rate was divided by the number of offspring in each chamber. The total number of individuals was used for eggs since viability is not reliably determinable until approximately 7 days post-oviposition [48] and eggs were put into chambers before day 7. Only the total number of successfully hatched larvae was used in calculating larval VCO_2_. Larval groups were inspected before and after metabolic measurements. Any dead larvae were documented and removed from calculations. All VCO_2_ (μl/h/ind) averages corrected for the number of offspring can be found in Table 1.

**Fig. 4:**
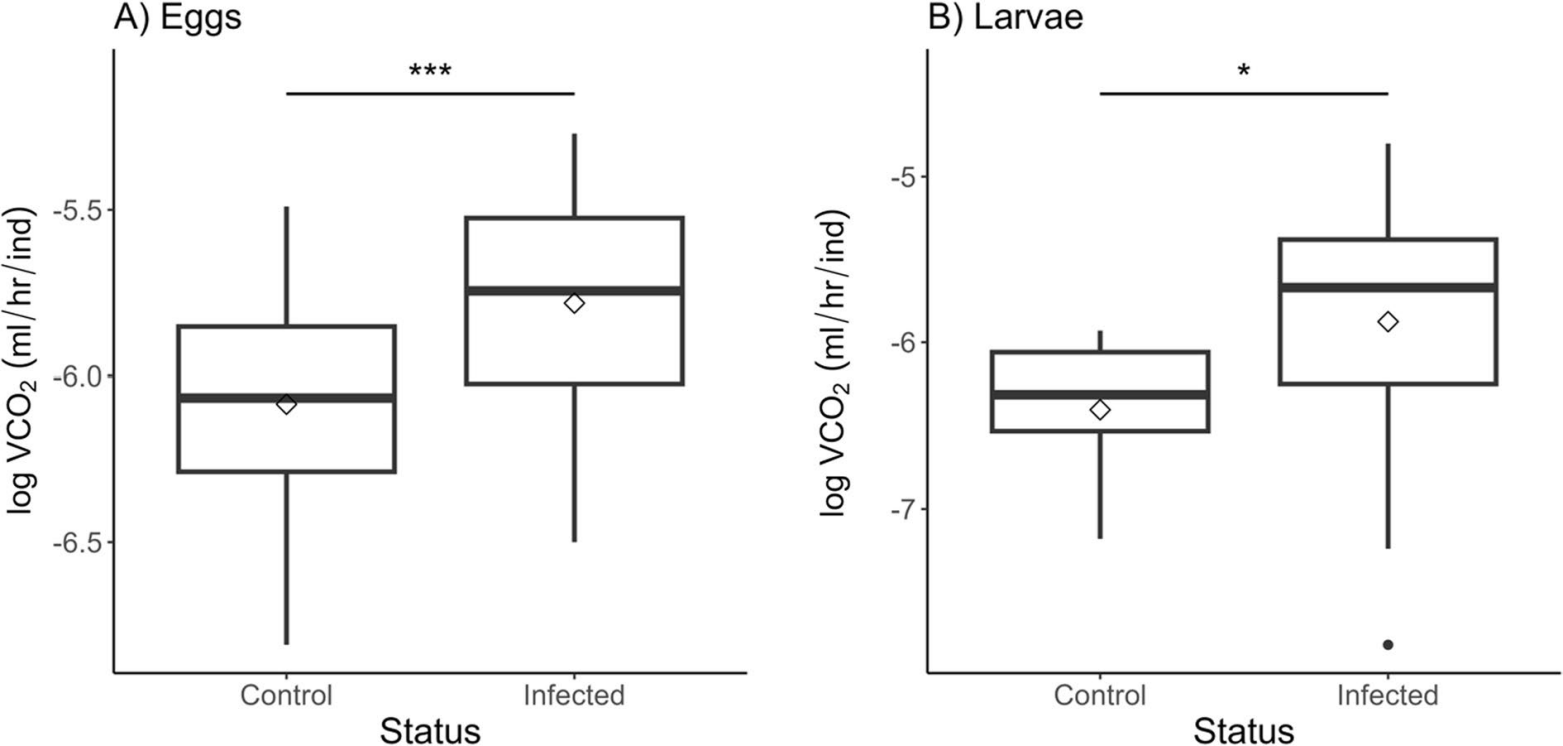
Metabolic rates for *R. microplus* eggs (**A**) and larvae (**B**). VCO_2_ was divided by the number of individuals in each chamber. Significant differences are denoted by * < 0.05, ** < 0.01, and *** *P* < 0.001. Linear mixed-effect model results can be found in Table 2. Extending lines represent the 25th to 75th percentiles of the data. Points beyond are considered outliers

There were no significant differences between weeks 1 and 2 when eggs were measured (linear mixed-effect model; *df* = 127, *t*-value = −0.33, *P* = 0.74); therefore, both weeks were combined for the analysis. Eggs from female *R. microplus* that fed on a calf infected with *B. bovis* had double the metabolic rate relative to eggs from uninfected ticks (Fig. 4A, Table 1). Similarly, larvae from infected female ticks had sixfold higher metabolic rates than controls (Fig. 4B, Table 1). Interestingly, control larvae had significantly lower metabolic rates than when they were eggs (linear mixed-effect model; *df* = 63, *t*-value = −3.373, *P* = 0.0004; Table 1). The same pattern was not seen for offspring from infected female ticks. Metabolic rates of infected offspring did not significantly change after eggs hatched into larvae (linear mixed-effect model; *df* = 101, *t*-value = −0.888, *P* = 0.377; Table 1).

### Hatching

**Fig. 5:**
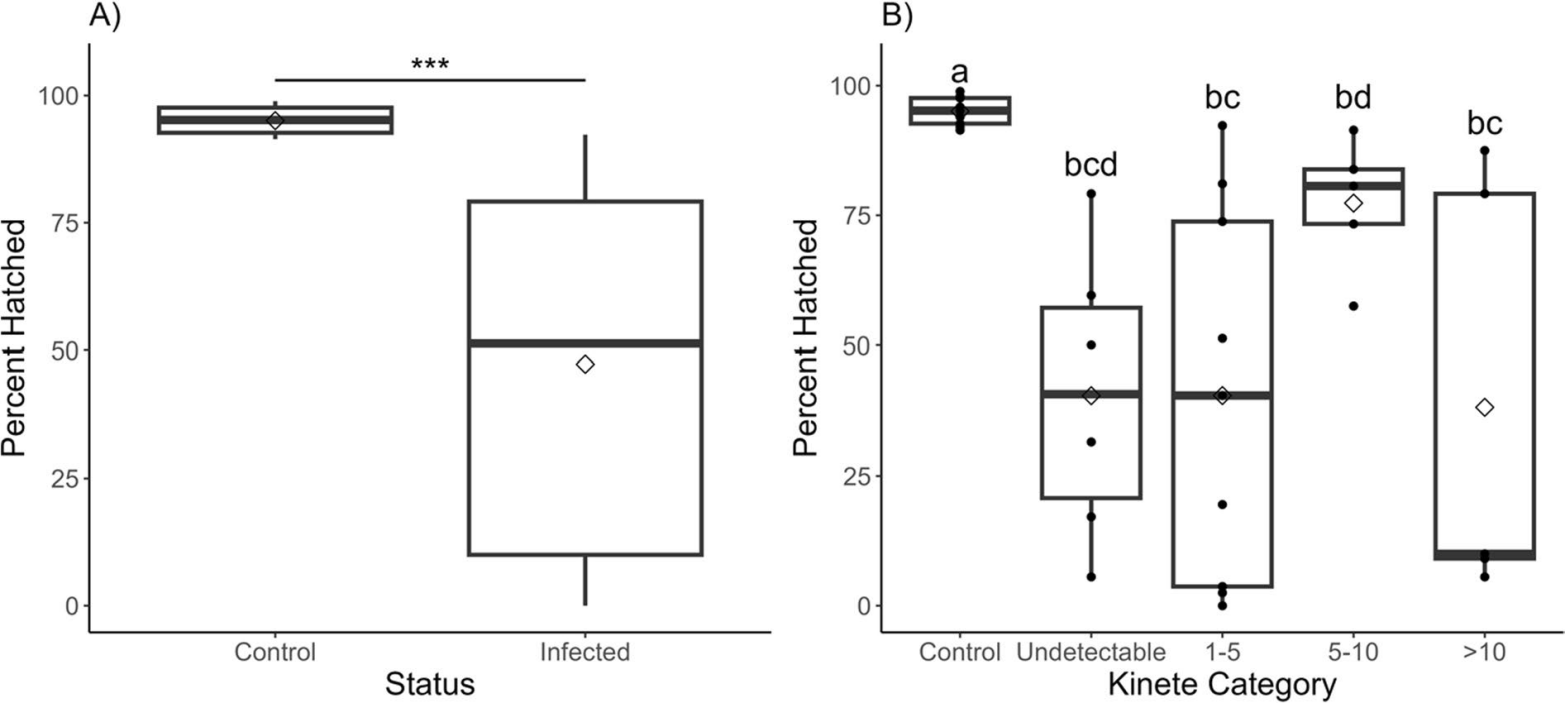
Percentage of larvae that successfully hatched. **A** More control offspring hatched compared to females that fed on an infected calf. Significant differences are denoted by * < 0.05, ** < 0.01, and *** *P* < 0.001. **B** Hatch percentages varied when offspring were separated based on the infection category of their corresponding maternal tick. Boxes with different letters are significantly different from each other (*P* < 0.05). Extending lines represent the 25th to 75th percentiles of the data. Points beyond are considered outliers

Control larvae (95.1%) were twice as likely to hatch as larvae from infected female ticks (45.5%; Table 3; Fig. 5A). Successful hatching percentages were highly variable for offspring from infected female ticks (0–92.3%). The range for control larvae was much narrower (91.4–98.9%). One group of control offspring was removed due to an exceptionally low hatching percentage (50%) that was determined to be an outlier (Dixon’s test: *Q* = 0.86, *P* < 0.0001). Offspring were separated into groups based on the number of kinetes measured in their corresponding maternal tick’s hemolymph smears (Fig. 5B). Overall, each category had a wide range of hatch percentages. Larvae from female ticks with 5–10 kinetes (77.4% ± 5.75; *n* = 5) had a higher likelihood of hatching from eggs than categories with 1–5 (40.5% ± 12.0; *n* = 9) and > 10 kinetes (37.2% ± 17.9; *n* = 5; Table 3; Fig. 5B).

**Table 3:** Results for a binomial generalized mixed-effect model used to determine the maximum likelihood of hatching, with tick ID as a random effect

	Model term	Estimate	Std error	*z*-value	*P*-value
*Hatching by treatment*					
Likelihood of hatching	Intercept	2.66	0.389	6.83	*P* < 0.0001
	Status (infected)	−2.99	0.263	−11.4	*P* < 0.0001
*Hatching by maternal kinete category*					
Likelihood of hatching	Intercept	2.83	0.367	7.70	*P* < 0.0001
	Undetectable	−3.13	0.413	−7.58	*P* < 0.0001
	1–5	−3.38	0.409	−8.25	*P* < 0.0001
	5–10	−1.62	0.494	−3.27	*P* = 0.0011
	> 10	−3.68	0.608	−6.05	*P* < 0.0001

## Discussion

Ticks play a key role in pathogen transmission to animals and humans, which could be better understood by investigating physiology at an organismal level. The effects of infection on tick metabolic rates are unknown, but whether pathogens can energetically benefit the tick or lead to deleterious consequences has major implications for infection spread and persistence within tick populations. Our hypothesis that the metabolic rate is altered across life stages when *R. microplus* are infected with *B. bovis* was supported. Replete female ticks infected with *B. bovis* had lower metabolic rates (Fig. 2A), while their offspring had higher metabolic rates as eggs and larvae (Fig. 4). Additionally, the body mass of infected replete females was reduced and their offspring had lower hatching success.

Adult replete females had approximately 25% reduction in body mass after feeding on an infected calf. Neither treatment group had significant changes in mass over the 3 days they were measured. While this study did not measure fecundity, reduction in mass has a direct negative relationship with fecundity in hematophagous arthropods, such as ticks [49]. Body mass was a strong predictor for VCO_2_ for infected replete females but not control ticks (Fig. 2C). *Rhipicephalus microplus* that fed on an uninfected calf had consistent metabolic rates despite differences in mass (Fig. 2C), and metabolic rates were generally higher than those of ticks that fed on an infected calf (Fig. 2B). These results suggest that uninfected females are respiring at maximum capacity in response to digestion and egg production. Females that fed on a *B. bovis*-infected calf exhibited a positive linear relationship between body mass and VCO_2_ with CO_2_ output increasing with body mass (Fig. 2C). This linear response suggests that infected female *R. microplus* are responding to infection differently and are potentially unable to maximally digest and convert their blood meals to eggs.

In a short period of time, replete females need to digest their final blood meal and begin egg development before oviposition. Adult replete females were measured for 3 days after drop-off to capture changes in metabolic rates over this period. VCO_2_ did not differ on day 1; however, on days 2 and 3, VCO_2_ was higher in female *R. microplus* that fed on an uninfected calf (Fig. 2B). VCO_2_ did not change over the 3 days for infected *R. microplus*. Our study only measured up to day 3 because numerous females began ovipositing on day 4, and oviposition has been shown to affect respiration rates in ticks [50]. Differences in VCO_2_ may become greater in individuals that had not yet started ovipositing. Decreased metabolic rates in response to infection are similar to other arthropods, such as *Drosophila* and mosquitoes [16, 18]; however, their offspring had the opposite response.

Embryogenesis requires metabolic pathways, such as glycolysis and gluconeogenesis, to be upregulated for development [51, 52]. Uninfected ticks were twice as likely to hatch as ticks from infected females (Fig. 5). This could reflect lower contributions of energetic reserves from infected mothers which is exacerbated by higher metabolic rates in offspring, resulting in egg mortality. An increased metabolic rate may indicate that infected ticks are utilizing their metabolic stores more rapidly. As resources are depleted, hatched ticks experience increases in metabolic rates and time to quest [53]. Since offspring from infected ticks have higher metabolic rates than controls (Fig. 4), larvae may begin searching for a host sooner to replenish energetic reserves. If infected larvae quest earlier than their uninfected counterparts, these larvae may infect naïve hosts that their uninfected siblings will later feed on. Naïve calves exposed to *B. bovis*-infected larvae become PCR-positive around 9 days post-larval attachment [54]. Peak parasitemia will occur between 9 and 15 days, and at this point, subsequent larvae that feed during this acute infection phase are more likely to acquire *B. bovis* [24]. Therefore, earlier questing by infected *R. microplus* larvae may lead to greater *B. bovis* transmission within a local population of ticks. Future research should investigate the effects of infection on metabolites, longevity, and questing behavior to determine whether higher metabolic rates in infected offspring result in greater transmission or lead to potentially deleterious impacts on infected ticks and limit overall fitness.

One explanation for suppressed metabolic rates in replete females infected with *B. bovis* compared with uninfected females is downregulation of certain genes associated with metabolism or energetically demanding processes. During infection with *B. bovis*, several genes in adult female *R. microplus* are significantly downregulated in response to infection [55]. These genes include those involved in immune responses, detoxification processes, and DNA replication. The downregulation may suppress the tick immune response and facilitate the replication and transmission of *B. bovis* to the tick ovaries and eggs [56]. In larvae, several genes are upregulated in response to infection including stress proteins, components of the Toll-like receptor pathway, and genes involved in oxidative stress response [57]. This may allow the larvae to deal with the physiological stress associated with infection and partly explain the increased metabolic rates observed in larvae.

The impacts of infection on metabolic rates are well studied in vertebrates; however, the influence on invertebrates is largely unexplored. For a pathogen and its vector, the physiological effects may be more complex. To be persistent, a pathogen must not kill its host or be too physiologically deleterious for the vector [58, 59]. Measuring metabolic rate and other physiological characteristics of additional tick species and their pathogens, especially newly emerging ticks and pathogens, will improve our understanding of how infection influences vector physiology and whether these relationships can be mutualistic or antagonistic. These generalized trends can be included in disease spread models, to predict disease outbreaks. For instance, *R. microplus* has been successfully eradicated from most of the United States and is now confined to southern Texas [28–30]; however, with factors such as climate change, wildlife host movement, and pesticide resistance, reintroduction into other states remains a looming threat [60–62]. In addition, the introduction and range expansion of new invasive tick species, such as *Haemaphysalis longicornis*, highlights the impact of globalization and increased trade on the spread of novel ticks and pathogens to new regions [63, 64]. With the rise in tick-borne diseases, future studies should investigate changes in physiology, ecology, and behavior at the tick–pathogen interface to better predict tick-borne disease spread and outbreaks.

## Supplementary Information

Supplementary material 1.

## Data Availability

The full dataset will be available from the Dryad digital repository upon peer review.
