# Supplementary material for: Infection with Babesia bovis alters metabolic rates of Rhipicephalus microplus ticks across life stages
Source: Parasit Vectors. 2025 Mar 1;18:81. doi: 10.1186/s13071-024-06645-3 (PMC11872307; doi:10.1186/s13071-024-06645-3)
Supplement: Supplementary file 1 — Supplementary material 1. [file 13071_2024_6645_MOESM1_ESM.docx]

**Supplemental Figures**


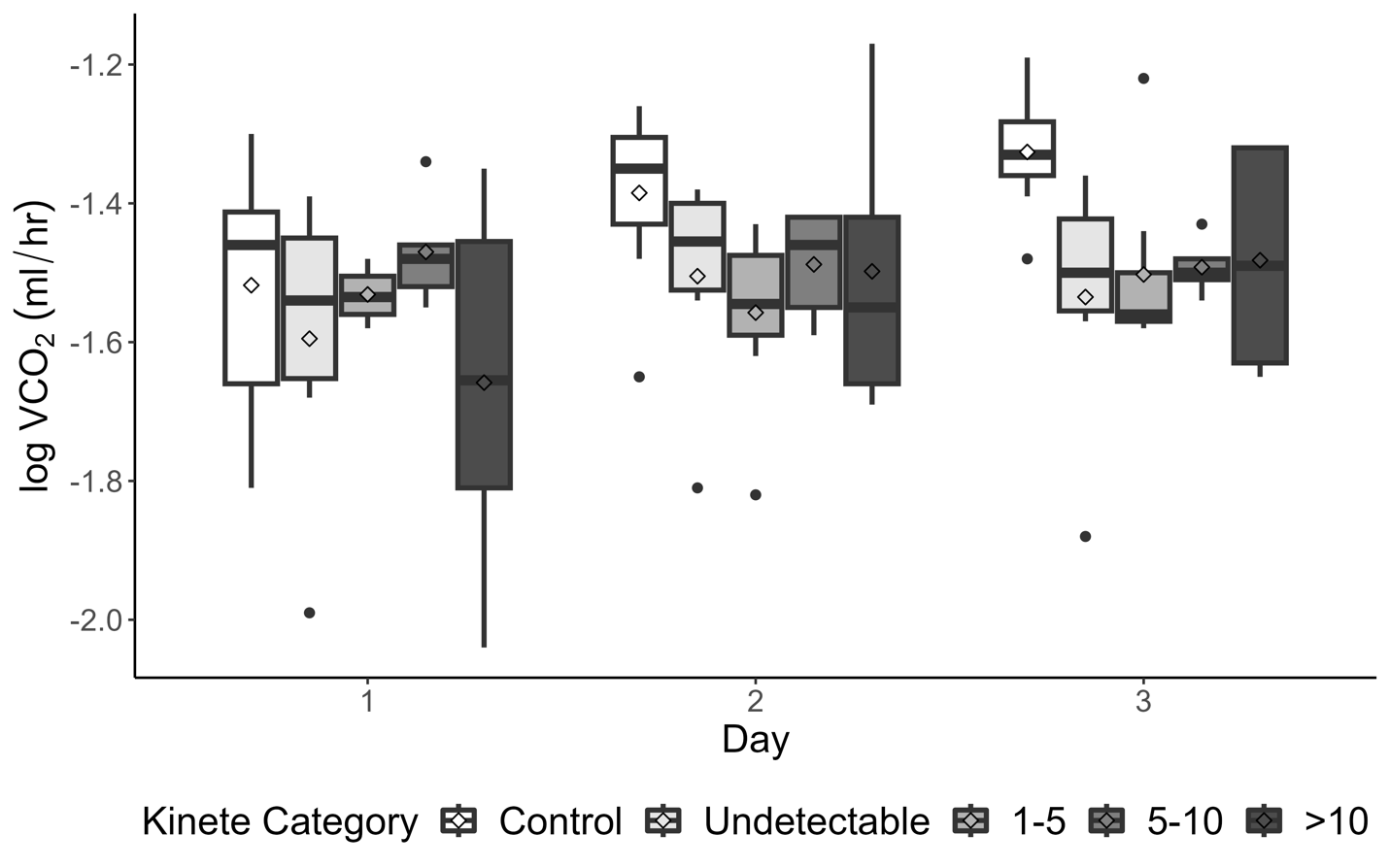


**Supplemental Figure 1.** Changes in VCO_2_ across days for each kinete category. Replete females were categorized based on the number of kinetes present in a hemolymph smear. Both kinete category and day were significant in a global generalized linear mixed-model with tick ID as a random effect. However, when investigating further, only ticks in the control and >10 kinete categories had changes in VCO_2_ across days and was significantly different from the other kinete categories. Output from all analyses can be found in Supplemental Table 2.

**Supplemental Table 1.** Average VCO_2_ (ml/hr) with SEM for each kinete category across the three days replete female R. microplus were measured.

|  | ***Day*** | | |
| --- | --- | --- | --- |
| ***Kinete Category*** | 1 | 2 | 3 |
| Control | 0.032 ± 0.004 | 0.043 ± 0.003 | 0.048 ± 0.003 |
| Undetectable | 0.028 ± 0.005 | 0.033 ± 0.004 | 0.031 ± 0.004 |
| 1-5 | 0.030 ± 0.001 | 0.029 ± 0.003 | 0.033 ± 0.004 |
| 5-10 | 0.034 ± 0.003 | 0.033 ± 0.002 | 0.032 ± 0.001 |
| >10 | 0.025 ± 0.006 | 0.036 ± 0.009 | 0.035 ± 0.006 |

**Supplemental Table 2.** Generalized linear-mixed models comparing absolute VCO_2_ of replete females based on the number of kinetes in their hemolymph smear and day measured. Tick ID was used as the random effect.

| ***Global Model*** | | | | | |  |
| --- | --- | --- | --- | --- | --- | --- |
|  | ***Model Term*** | ***Estimate*** | ***Std error*** | ***t value*** | ***p value*** |  |
| Absolute VCO_2_ | Intercept | -1.52 | 0.04 | -35.8 | p < 0.0001 |  |
|  | Undetectable | -0.09 | 0.05 | -1.89 | p = 0.063 |  |
|  | 1-5 kinetes | -0.12 | 0.04 | -2.91 | p = 0.005 |  |
|  | 5-10 kinetes | -0.08 | 0.06 | -1.39 | p = 0.168 |  |
|  | >10 kinetes | -0.11 | 0.05 | -2.03 | p = 0.046 |  |
|  | Day | 0.05 | 0.01 | 3.52 | p = 0.0001 |  |
| ***Day 1*** | | | | | |  |
|  | ***Model Term*** | ***Estimate*** | ***Std error*** | ***t value*** | ***p value*** |  |
| Absolute VCO_2_ | Intercept | -1.52 | 0.054 | -27.9 | p < 0.0001 |  |
|  | Undetectable | -0.08 | 0.089 | -0.87 | p = 0.450 |  |
|  | 1-5 kinetes | -0.01 | 0.082 | -0.16 | p = 0.881 |  |
|  | 5-10 kinetes | 0.05 | 0.094 | 0.51 | p = 0.646 |  |
|  | >10 kinetes | -0.14 | 0.089 | -1.58 | p = 0.213 |  |
| ***Day 2*** | | | | | |  |
|  | ***Model Term*** | ***Estimate*** | ***Std error*** | ***t value*** | ***p value*** |  |
| Absolute VCO_2_ | Intercept | -1.40 | 0.042 | -33.0 | p < 0.0001 |  |
|  | Undetectable | -0.10 | 0.066 | -1.55 | p = 0.218 |  |
|  | 1-5 kinetes | -0.16 | 0.059 | -2.76 | p = 0.070 |  |
|  | 5-10 kinetes | -0.09 | 0.073 | -1.29 | p = 0.288 |  |
|  | >10 kinetes | -0.08 | 0.073 | -1.04 | p = 0.375 |  |
| ***Day 3*** | | | | | |  |
|  | ***Model Term*** | ***Estimate*** | ***Std error*** | ***t value*** | ***p value*** |  |
| Absolute VCO_2_ | Intercept | -1.33 | 0.039 | -34.0 | p < 0.0001 |  |
|  | Undetectable | -0.21 | 0.063 | -3.28 | p = 0.047 |  |
|  | 1-5 kinetes | -0.18 | 0.058 | -3.07 | p = 0.054 |  |
|  | 5-10 kinetes | -0.16 | 0.067 | -2.45 | p = 0.092 |  |
|  | >10 kinetes | -0.16 | 0.067 | -2.21 | p = 0.105 |  |
| ***Kinete Category: Control*** | | | | | |  |
|  | ***Model Term*** | ***Estimate*** | ***Std error*** | ***t value*** | ***p value*** |  |
| Absolute VCO_2_ | Intercept | -1.60 | 0.057 | -28.2 | p < 0.0001 |  |
|  | Day | 0.10 | 0.024 | 3.93 | p < 0.0001 |  |
| ***Kinete Category: Undetectable*** | | | | | |  |
|  | ***Model Term*** | ***Estimate*** | ***Std error*** | ***t value*** | ***p value*** |  |
| Absolute VCO_2_ | Intercept | -1.61 | 0.097 | -16.6 | p < 0.0001 |  |
|  | Day | 0.03 | 0.035 | 0.85 | p = 0.416 |  |
| ***Kinete Category: 1-5*** | | | | | |  |
|  | ***Model Term*** | ***Estimate*** | ***Std error*** | ***t value*** | ***p value*** |  |
| Absolute VCO_2_ | Intercept | -1.56 | 0.052 | -29.9 | p < 0.0001 |  |
|  | Day | 0.01 | 0.023 | 0.63 | p = 0.535 |  |
| ***Kinete Category: 5-10*** | | | | | | |
|  | | ***Model Term*** | ***Estimate*** | ***Std error*** | ***t value*** | ***p value*** |
| Absolute VCO_2_ | | Intercept | -1.46 | 0.036 | -40.1 | p < 0.0001 |
|  |  | Day | -0.01 | 0.012 | -0.90 | p = 0.392 |
| ***Kinete Category: >10*** | | | | | | |
|  | | ***Model Term*** | ***Estimate*** | ***Std error*** | ***t value*** | ***p value*** |
| Absolute VCO_2_ | | Intercept | -1.72 | 0.102 | -16.8 | p < 0.0001 |
|  |  | Day | 0.07 | 0.031 | 2.40 | p = 0.040 |
